# Depression and Anxiety Among Sexual Minorities in the United States: A Cross-Sectional Analysis of the National Health Interview Survey

**DOI:** 10.7759/cureus.64580

**Published:** 2024-07-15

**Authors:** Anna L Miller, Larry E Miller, Mehul Bhattacharyya, Ruemon Bhattacharyya

**Affiliations:** 1 Clinical Research, Miller Scientific, Johnson City, USA; 2 Data Analytics, Arizona State University, Tempe, USA; 3 Public Affairs and Economics, University of California Los Angeles, Los Angeles, USA

**Keywords:** nhis, mental health, lgbtq, lesbian, homosexual, heterosexual, gay, depression, bisexual, anxiety

## Abstract

Background and objective

Sexual minorities experience large-scale mental health disparities, yet recent national data on them remains scarce. This study aimed to examine the prevalence of depressive and anxiety symptoms by sexual orientation in a nationally representative sample of adults in the United States (US).

Methods

This cross-sectional analysis utilized data from 26,311 adults in the 2022 National Health Interview Survey. Sexual orientation was classified as sexual minority (gay/lesbian, bisexual, or other non-heterosexual identity) or heterosexual. Self-reported depression was assessed using the eight-item Patient Health Questionnaire (PHQ-8), and anxiety was evaluated using the seven-item Generalized Anxiety Disorder Scale (GAD-7). Logistic regression models were employed to compare mental health outcomes between sexual minority and heterosexual respondents.

Results

Sexual minorities comprised 6.6% of the weighted sample. Compared to heterosexuals, sexual minorities were younger, more often female, unmarried, and had higher poverty despite greater employment (all p<0.001). Approximately half of sexual minorities screened positive for depression (49.0%) and anxiety (44.3%), compared to 19.5% and 16.4% of heterosexuals, respectively. After covariate adjustment, sexual minorities had over three-fold higher odds of depression [odds ratio (OR): 3.27; 95% confidence interval (CI): 2.86-3.73] and anxiety (OR: 2.97; 95% CI: 2.57-3.42). The prevalence was highest among sexual minority youth, with depression in 54.9-61.1% and anxiety in 49.0-59.2%, depending on income levels.

Conclusions

In this nationally representative study, sexual minorities demonstrated a high burden of depression and anxiety symptoms compared to heterosexuals. Sexual orientation independently predicted mental health disparities beyond other sociodemographic characteristics. Targeted interventions are recommended to address psychiatric disease disparities that disproportionately impact vulnerable sexual minority subgroups.

## Introduction

Depression and anxiety disorders are highly prevalent mental health conditions and leading causes of disability worldwide [1,2]. Sexual minorities - comprising individuals who identify as lesbian, gay, bisexual, transgender, or other non-heterosexual orientations - may experience higher rates of depressive and anxiety symptoms compared to their heterosexual counterparts [3,4]. According to the minority stress theory [5], sexual minorities encounter unique psychosocial stressors, such as widespread interpersonal and structural stigma, institutional discrimination, and inadequate social support networks, which may exacerbate their vulnerability to adverse mental health outcomes.

Additionally, sexual minorities face substantial barriers to accessing and engaging with mental healthcare services [6]. Experiencing discrimination [7] and encountering limited cultural competency [8] may deter sexual minorities from seeking care. Without proper mental health treatment, sexual minorities suffer from increased risks of suicide, lower quality of life, functional impairments, and chronic psychiatric conditions [9]. Furthermore, those receiving treatment report lower satisfaction levels, poorer therapeutic relationships, and a higher likelihood of early termination [4].

Documenting population-based mental health disparities is crucial for identifying high-risk groups, raising awareness, and informing public policy to promote health equity. Healthy People 2030 is a national initiative in the United States (US) with 10-year targets/objectives to improve the health and well-being of Americans, aiming to address health disparities and promote overall public health. A key objective of Healthy People 2030 is expanding national surveillance efforts to better characterize lesbian, gay, bisexual, transgender, and queer/questioning (LGBTQ+) health disparities [10]. Yet, despite these efforts, recent population-based data characterizing mental health among sexual minorities remain limited. To address this gap, the current study aimed to examine the prevalence of depressive and anxiety symptoms in sexual minorities among a nationally representative sample of adults in the US. We hypothesized that sexual minorities would have a higher prevalence of depressive and anxiety symptoms than heterosexuals, even after adjusting for sociodemographic and clinical confounders.

## Materials and methods

Study design and participants

This cross-sectional study used the 2022 National Health Interview Survey (NHIS) data. The NHIS is an annual household interview survey that uses geographically clustered sampling techniques involving households, with one adult chosen randomly from each household. Active-duty military personnel, civilians living on military bases, persons in long-term care and correctional facilities, and persons with no fixed household address were excluded. Interviews were conducted between January 1 and December 31, 2022. The study sample included 27,651 adults (47.7% response rate). Informed consent was obtained from all participants, and the National Centers for Health Statistics Ethics Review Committee approved the study. Secondary analyses of these publicly accessible de-identified data were exempt from Institutional Review Board review per the US Department of Health and Human Services Policy for Protection of Human Research Subjects (45 CFR 46.101(b)(4)). This study adhered to the Strengthening the Reporting of Observational Studies in Epidemiology (STROBE) reporting guidelines [11].

Sexual orientation classification

Sexual orientation was broadly classified as sexual minority or heterosexual for the primary analyses. Individuals were asked, "Do you think of yourself as a) gay/lesbian, b) straight, that is, not gay/lesbian, c) bisexual, d) something else, or e) you don't know the answer"? Individuals reporting their sexual orientation as gay, lesbian, bisexual, "something else," or "I don't know" were considered sexual minorities.

Outcomes

The primary outcomes of this study were self-reported depression and anxiety. Depression was assessed using the Patient Health Questionnaire (PHQ-8), an eight-item self-assessment of depressive symptoms in the past two weeks. Each item was scored from 0 (“not at all”) to 3 (“nearly every day”), providing a total scoring range of 0-24 points. The severity of depressive symptoms was classified as none/minimal (scores 0-4), mild (scores 5-9), moderate (scores 10-14), or severe (scores 15-24) [12]. Participants with PHQ-8 scores of 5 or greater were classified as having depressive symptoms in the primary analysis. We performed a sensitivity analysis using a cutoff score of 10 or greater, indicating probable major depressive disorder with high sensitivity (>99%) and specificity (92%) [13].

Anxiety was evaluated using the seven-item Generalized Anxiety Disorder Scale (GAD-7), a self-report questionnaire that assesses anxiety symptoms over the previous two weeks [14]. Each item was rated from 0 (“not at all”) to 3 (“nearly every day”), providing a total scoring range of 0-21 points. The severity of anxiety symptoms was classified as none/minimal (scores 0-4), mild (scores 5-9), moderate (scores 10-14), or severe (scores 15-21). The primary analysis classified participants with GAD-7 scores of 5 or greater as having anxiety symptoms. We performed a sensitivity analysis using a cutoff score of 10 points or greater, which indicates a probable general anxiety disorder with high sensitivity (89%) and specificity (82%) [14].

Additional assessments related to mental health included medication use for depression, anxiety, or sleep, receiving mental health counseling, difficulty with remembering/concentrating, and difficulty engaging in social activities. Sociodemographic characteristics included age, sex, race, marital status, employment, and income as a percentage of the federal poverty guidelines. Physical health characteristics included variables with a prevalence of at least 5% in either of the sexual orientation groups and included obesity, hypertension, asthma, high cholesterol, arthritis, diabetes, cancer, lung disease, and coronary heart disease.

Data analysis

The study used complex sample analysis methods that accounted for sampling weights, sample strata, and clustering to provide accurate parameter estimates for the US population. Sample weights were calculated as the inverse of the probability of selection, with adjustments for nonresponse patterns, to estimate the number of individuals in the population represented by each survey respondent. Taylor series linearization was used to estimate the variance, accounting for the stratified cluster sampling design. We used a chi-squared test to compare subject characteristics between sexual minority and heterosexual groups. The mental health characteristics of sexual minorities and heterosexuals were compared using univariable and multivariable logistic regression models adjusted for sociodemographic and physical health characteristics.

To assess the relative importance of variables in the multivariable logistic regression models, we employed a machine learning approach termed SHapley Additive exPlanations (SHAP). SHAP is a fully adjusted multivariable technique that comprehensively evaluates the relative contribution of each independent variable while considering all possible interactions among them. For each model, SHAP values were computed for each independent variable, providing a quantitative measure of their importance ranging from 0% to 100% [15]. This technique determined the relative importance of predictors of depression and anxiety using cutoffs of 5 points or higher (mild to severe symptoms) on the PHQ-8 and GAD-7 scales, respectively. Sensitivity analyses were also performed at cutoffs of 10 points or higher (indicating moderate to severe symptoms) on each scale. Statistical analyses were performed using Stata v18 (StataCorp, College Station, TX).

## Results

Sexual orientation data were obtained from 26,311 (95.2%) adults in the 2022 National Health Interview Survey, of whom 1,578 (6.0%) identified as a sexual minority. In the adjusted analysis, sexual minorities comprised 6.6% of the adult US population. Within the sexual minority group, the reported sexual orientations included bisexual (39.6%), gay/lesbian (30.9%), "I don't know" (16.9%), and "Something else" (12.6%).

Compared with their heterosexual counterparts, sexual minority adults were younger (58.5% vs. 26.5% aged 18-34 years), more likely to be female (61.0% vs. 50.6%), and less likely to be married (22.9% vs. 53.6%) (all p<0.001). A higher proportion of sexual minorities had incomes <200% of the federal poverty level (32.5% vs. 26.9%) despite higher levels of employment (86.3% vs. 80.3%) (both p<0.001). Sexual minorities had a lower prevalence of most physical health conditions, including hypertension, high cholesterol, arthritis, diabetes, cancer, and coronary heart disease (all p<0.001), but had higher rates of asthma (22.4% vs. 14.0%, p<0.001) (Table 1).

**Table 1:** Characteristics of adults in the 2022 National Health Interview Survey by sexual orientation AIAN: American Indian and Alaskan Native; FPG: federal poverty guidelines; Hetero: heterosexual; SM: sexual minority

Variables	SM	Hetero	P-value
Sample data			
Number of respondents	1,578	24,733	
Percentage of respondents (unadjusted)	6.0	94.0	
Percentage of respondents (adjusted)	6.6	93.4	
Sexual orientation, %			
Bisexual	39.6	0.0	
Gay/lesbian	30.9	0.0	
I don't know	16.9	0.0	
Something else	12.6	0.0	
Straight	0.0	100.0	
Sociodemographic characteristics, %			
Age (years)			<0.001
18-34	58.5	26.5	
35-64	32.7	50.2	
≥65	8.8	23.2	
Female sex	61.0	50.6	<0.001
Race			<0.001
White	77.7	77.1	
Black	11.4	12.8	
Asian	4.6	6.7	
AIAN	1.4	1.2	
Other/multirace	4.9	2.3	
College degree	32.6	32.9	0.87
Marital status			<0.001
Married	22.9	53.6	
Partner	15.4	8.4	
Single	61.7	37.9	
Employed	86.3	80.3	<0.001
Income			<0.001
<200% FPG	32.5	26.9	
200-399% FPG	29.2	29.1	
≥400% FPG	38.3	44.1	
Physical health characteristics, %			
Obesity	34.3	33.2	0.45
Hypertension	22.5	32.8	<0.001
Asthma	22.4	14.0	<0.001
High cholesterol	19.2	28.0	<0.001
Arthritis	16.4	22.0	<0.001
Diabetes	6.5	9.8	<0.001
Cancer	6.2	9.9	<0.001
Lung disease	4.0	4.6	0.38
Coronary heart disease	2.6	5.2	<0.001

**Figure 1:**
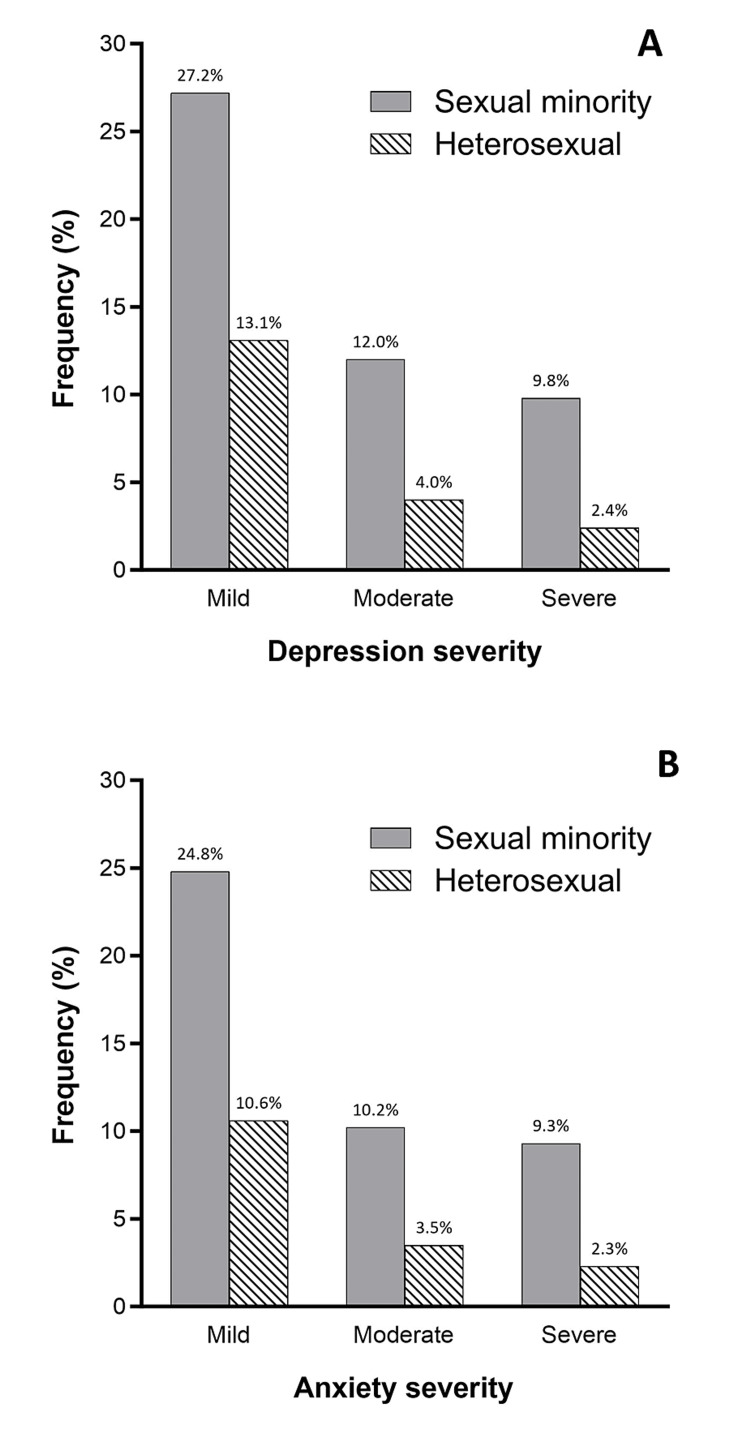
Severity of self-reported depression (A) and anxiety (B) by sexual orientation among adults in the 2022 National Health Interview Survey Comparing sexual minorities to heterosexuals, the frequency of self-reported depression assessed using the Patient Health Questionnaire-8 scale was 49.0% vs. 19.5%, and the frequency of self-reported anxiety assessed using the Generalized Anxiety Disorder-7 scale was 44.3% vs. 16.4%

Mental health disparities were significant between sexual minority and heterosexual adults. Sexual minorities had four-fold higher odds of screening positive for depression [odds ratio (OR): 3.97; 95% confidence interval (CI): 3.51-4.50] and anxiety disorders (OR: 4.06; 95% CI: 3.57-4.61) compared with heterosexuals. Approximately half of the sexual minorities screened positive for depression (49.0%) and anxiety (44.3%), compared to 19.5% and 16.4% of heterosexuals, respectively. The higher prevalence of depression and anxiety in sexual minorities was observed across all levels of severity (Figure 1).

Medication use for depression, anxiety, and sleep difficulties was also more prevalent among sexual minorities. One-third of sexual minorities (33.5%) reported receiving mental health counseling in the past year, compared to only 11.2% of heterosexuals. Over one-quarter of sexual minorities reported difficulties participating in social activities (27.4% vs. 10.2% of heterosexuals) and remembering/concentrating (36.2% vs. 19.1%) (Table 2).

**Table 2:** Mental health characteristics of adults in the 2022 National Health Interview Survey by sexual orientation *Defined as a PHQ-8 scale score of 5 or greater [13]. **Defined as a GAD-7 scale score of 5 or greater [14]. ***Model adjusted for all covariates listed in Table 1 CI: confidence interval; GAD-7: seven-item Generalized Anxiety Disorder Scale; Hetero: heterosexual; PHQ-8: eight-item Patient Health Questionnaire; SM: sexual minority

Variables	SM	Hetero	Unadjusted model			Fully adjusted model***		
			Odds ratio	95% CI	P-value	Odds ratio	95% CI	P-value
Depression, %*	49.0	19.5	3.97	3.51, 4.50	<0.001	3.27	2.86, 3.73	<0.001
Take medication for depression, %	24.5	10.8	2.70	2.33, 3.13	<0.001	2.49	2.11, 2.94	<0.001
Anxiety, %**	44.3	16.4	4.06	3.57, 4.61	<0.001	2.97	2.57, 3.42	<0.001
Take medication for anxiety, %	27.5	13.3	2.48	2.14, 2.88	<0.001	2.19	1.85, 2.60	<0.001
Received mental health counseling in the past 12 months, %	33.5	11.2	4.01	3.50, 4.59	<0.001	2.82	2.43, 3.28	<0.001
Difficulty remembering/concentrating, %	36.2	19.1	2.41	2.11, 2.76	<0.001	2.53	2.18, 2.95	<0.001
Difficulty participating in social activities, %	27.4	10.2	3.33	2.85, 3.88	<0.001	3.18	2.65, 3.82	<0.001
Take medication for sleep, %	27.9	18.6	1.69	1.48, 1.93	<0.001	1.63	1.40, 1.89	<0.001

In the univariable analyses, identifying as a sexual minority was the strongest predictor of depression (OR: 3.97; 95% CI: 3.51-4.50) (Table 3) and anxiety (OR: 4.06; 95% CI: 3.57-4.61) (Table 4) among the other sociodemographic and physical health characteristics. Younger age, female sex, lower education and income, lack of employment, and being unmarried were also associated with higher odds of depression and anxiety. Among the physical health conditions, arthritis, asthma, and lung disease had the strongest associations with increased odds of depression and anxiety.

**Table 3:** Univariable predictors of depression among adults in the 2022 National Health Interview Survey* *Depression defined as a PHQ-8 score of 5 or greater AIAN: American Indian and Alaskan Native; CI: confidence interval; FPG: federal poverty guidelines; PHQ-8: eight-item Patient Health Questionnaire

Variables	Unit of measure	Odds ratio	95% CI	P-value
Sexual minority	Yes vs. no	3.97	3.51, 4.50	<0.001
Age	Per 10-year decrease	1.09	1.07, 1.11	<0.001
Sex	Female vs. male	1.48	1.38, 1.58	<0.001
Race	Other/multirace vs. Asian	2.56	1.97, 3.32	<0.001
	AIAN vs. Asian	2.36	1.64, 3.38	
	Black vs. Asian	1.85	1.52, 2.26	
	White vs. Asian	1.80	1.52, 2.13	
College degree	No vs. yes	1.49	1.38, 1.60	<0.001
Marital status	Single vs. married	1.88	1.75, 2.03	<0.001
	Partner vs. married	1.77	1.57, 2.01	
Employed	No vs. yes	1.41	1.31, 1.52	<0.001
Income	<200% vs. ≥400% FPG	2.27	2.08, 2.48	<0.001
	200-399% vs. ≥400% FPG	1.56	1.43, 1.71	
Obesity	Yes vs. no	1.60	1.49, 1.71	<0.001
Hypertension	Yes vs. no	1.40	1.31, 1.51	<0.001
Asthma	Yes vs. no	2.06	1.88, 2.25	<0.001
High cholesterol	Yes vs. no	1.32	1.23, 1.42	<0.001
Arthritis	Yes vs. no	2.07	1.92, 2.24	<0.001
Diabetes	Yes vs. no	1.74	1.57, 1.93	<0.001
Cancer	Yes vs. no	1.27	1.14, 1.41	<0.001
Lung disease	Yes vs. no	3.08	2.70, 3.51	<0.001
Coronary heart disease	Yes vs. no	1.94	1.72, 2.19	<0.001

**Table 4:** Univariable predictors of anxiety among adults in the 2022 National Health Interview Survey* *Anxiety defined as a GAD-7 score of 5 or greater AIAN: American Indian and Alaskan Native; CI: confidence interval; FPG: federal poverty guidelines; GAD-7: seven-item Generalized Anxiety Disorder Scale

Variables	Unit of measure	Odds ratio	95% CI	P-value
Sexual minority	Yes vs. no	4.06	3.57, 4.61	<0.001
Age	Per 10-year decrease	1.22	1.19, 1.25	<0.001
Sex	Female vs. male	1.57	1.45, 1.69	<0.001
Race	Other/multirace vs. Asian	2.48	1.88, 3.27	<0.001
	AIAN vs. Asian	2.12	1.44, 3.13	
	Black vs. Asian	1.63	1.32, 2.01	
	White vs. Asian	1.62	1.35, 1.95	
College degree	No vs. yes	1.33	1.23, 1.45	<0.001
Marital status	Single vs. married	1.80	1.66, 1.94	<0.001
	Partner vs. married	2.05	1.78, 2.35	
Employed	No vs. yes	1.05	0.96, 1.15	0.32
Income	<200% vs. ≥400% FPG	2.07	1.90, 2.27	<0.001
	200-399% vs. ≥400% FPG	1.51	1.38, 1.66	
Obesity	Yes vs. no	1.34	1.24, 1.45	<0.001
Hypertension	Yes vs. no	1.06	0.98, 1.14	0.15
Asthma	Yes vs. no	2.02	1.83, 2.22	<0.001
High cholesterol	Yes vs. no	1.08	1.00, 1.17	0.06
Arthritis	Yes vs. no	1.54	1.42, 1.67	<0.001
Diabetes	Yes vs. no	1.27	1.13, 1.43	<0.001
Cancer	Yes vs. no	0.98	0.88, 1.11	0.79
Lung disease	Yes vs. no	2.51	2.20, 2.87	<0.001
Coronary heart disease	Yes vs. no	1.26	1.10, 1.44	0.001

**Figure 2:**
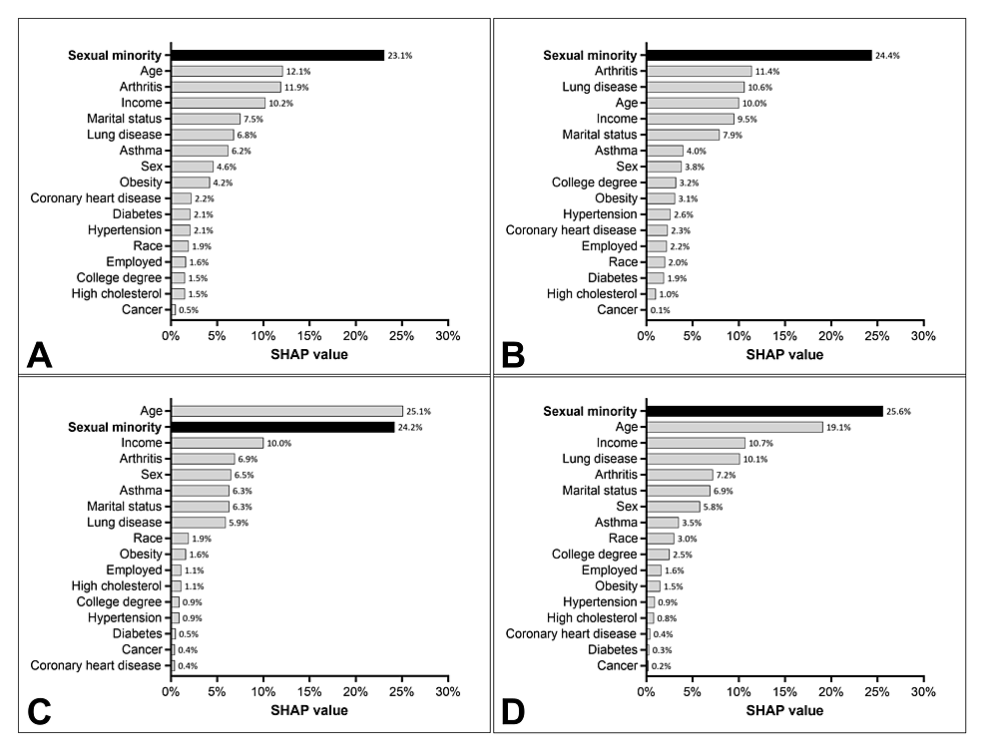
SHAP analysis SHAP values indicating the relative importance of covariates in a multivariable logistic regression model to predict (A) depression (mild to severe), (B) depression (moderate to severe), (C) anxiety (mild to severe), (D) anxiety (moderate to severe) among adults in the 2022 National Health Interview Survey SHAP: SHapley Additive exPlanations

In the fully-adjusted multivariable SHAP analyses, sexual minority status consistently emerged as one of the strongest predictors of depression and anxiety across all models, while age and income also received high relative importance scores (Figure 2).

The subgroups at the highest risk were lower-income adults aged 18-34 years, among whom depression was identified in 54.9-61.1% of sexual minorities and 17.9-23.8% of heterosexuals, depending on income class. Similarly, in young adults, anxiety was reported in 49.0-59.2% of sexual minorities, with higher values in lower-income individuals versus 17.9-23.7% in heterosexuals (Table 5).

**Table 5:** Prevalence of depression and anxiety in high-risk subgroups in the 2022 National Health Interview Survey *Defined as a PHQ-8 scale score of 5 or greater. **Defined as a GAD-7 scale score of 5 or greater FPG: federal poverty guidelines; GAD-7: seven-item Generalized Anxiety Disorder Scale; Hetero: heterosexual; PHQ-8: eight-item Patient Health Questionnaire SM: sexual minority

Agen (years)	Income	Depression (%)*		Anxiety (%)**	
		SM	Hetero	SM	Hetero
18-34	<200% FPG	61.1	23.1	56.9	22.0
	200-399% FPG	59.3	23.8	59.2	23.7
	≥400% FPG	54.9	17.9	49.0	17.9
35-64	<200% FPG	54.9	30.8	42.5	26.4
	200-399% FPG	38.2	19.1	33.6	16.5
	≥400% FPG	27.8	13.2	26.5	11.5
≥65	<200% FPG	31.6	25.4	19.0	15.2
	200-399% FPG	39.2	19.4	22.8	11.2
	≥400% FPG	16.4	12.5	8.3	7.9

## Discussion

In this cross-sectional analysis of a large, nationally representative sample of US adults, it was found that sexual minorities experienced a significantly higher burden of depression and anxiety than their heterosexual peers. Approximately half of sexual minorities screened positive for depressive and anxiety symptoms, four-fold higher than heterosexual respondents. These findings align with prior studies documenting mental health disparities related to sexual orientation minority status [3,16,17]. The prevalence of depression and anxiety among sexual minorities observed here mirrors estimates from previous national and international surveys [18]. The novelty of the current study is the use of the most recent NHIS data released in June 2023, providing the most contemporary data available on the mental health of sexual minorities. Additionally, this is the first population-based study to identify sexual minority status as the most important predictor of depression and anxiety compared to other sociodemographic and health characteristics.

Notably, identifying as a sexual minority was the strongest single predictor of depression and anxiety symptoms in the regression models. This highlights that sexual orientation, beyond confounding demographic and clinical variables, represents a strong independent risk factor for mood and anxiety disorders. Though research on underlying mechanisms remains limited, minority stress processes, including internalized stigma and identity concealment, may drive this association [19]. Within sexual minorities, depression and anxiety symptoms were most prevalent among low-income younger adults, suggesting that this subgroup faces particularly high psychiatric risks. It is plausible that minority stress from heterosexism and cissexism intensifies mental health issues already present in adolescence and early adulthood [20], possibly because experiences of discrimination peak during emerging adulthood [21]. Limited access to LGBTQ-affirming care and family estrangement can isolate some low-income sexual and gender minority youth, increasing mental health risks [22]. Financial instability may also strain mental health, and low-income sexual minorities experience more chronic stress from work/housing issues [23]. The convergence of developmental vulnerability, minority stress, and economic challenges may explain the high psychiatric disease burden among low-income young sexual minorities.

Our findings highlight the mental health disparities faced by sexual minorities, which align with minority stress theory [24], which posits that sexual minorities face unique psychosocial stressors, including interpersonal and structural stigma, discrimination, and social isolation, that confer a greater risk for mental health problems. Notably, a higher prevalence of sexual minority identification was reported among younger adults, which may reflect increased social acceptance and evolving attitudes towards diverse sexual orientations in recent decades. Furthermore, we found that younger age, lower socioeconomic status, unmarried status, and lack of social/occupational engagement increased the odds of depression and anxiety symptoms among sexual minorities. These sociodemographic factors may reflect or increase exposure to minority stress. The high prevalence of recent mental health counseling and psychiatric medication use, while possibly indicating appropriate treatment engagement, also suggests unmet needs and barriers to accessing high-quality mental healthcare [6]. To address mental health disparities, culturally competent mental healthcare is essential for sexual minorities to mitigate minority stress and promote well-being [25].

Targeted interventions may help address the higher rates of depression and anxiety among sexual minorities [26]. While mental health initiatives should support sexual minorities across demographic spectrums, prioritizing subgroups facing the greatest disparities may be warranted based on the evidence from this study indicating a higher disease burden. A targeted approach toward young, low-income LGBTQ+ populations may aim to address urgent needs efficiently but should occur alongside broader efforts to foster inclusivity for all sexual orientations and gender identities. Establishing free mental health clinics and support groups specifically for LGBTQ+ youth in underserved communities could increase access to care. Additionally, implementing educational campaigns and nondiscrimination policies in workplaces and schools may help to reduce stigma. By incorporating supportive policies and initiatives focusing on the most vulnerable subgroups, mental health disparities among sexual minorities may be reduced, aligning with Healthy People 2030 priorities [10].

Strengths and limitations

This population-based study had several important strengths. Firstly, the results were generalizable to the US population of non-institutionalized civilian adults. Second, the NHIS evaluated population-based health characteristics across different sociodemographic groups that may not be accurately estimated in smaller studies. Third, we determined the relative importance of covariates in predicting depression and anxiety using SHAP values, which enabled the practical interpretation of the regression model outputs [15]. Finally, the objectives of this study reflect those of Healthy People 2030 [10], underscoring the societal importance of the results.

However, there are some notable limitations as well. Primarily, the cross-sectional design precludes determining causal relationships or temporal associations between sexual orientation, minority stress, and mental health over time. Longitudinal analyses are needed to elucidate prospective associations between minority stress and mental health outcomes. Second, screening questionnaires without clinician confirmation may have misclassified diagnoses of depression and anxiety. Third, binary categorization of sexual orientation overlooked diversity and fluidity within LGBTQ+ populations, yet the sample sizes within each specific sexual orientation group were too small to support robust statistical analyses. This may have overlooked specific identity experiences and mental health profiles that differentiate specific sexual orientations. Fourth, sample bias due to non-participation and misclassification from reliance on self-report must be acknowledged as potential risks. Finally, contextual data were unavailable on factors like community connectivity, openness about sexual orientation identity, and early life experiences [27] that may mediate the observed associations.

## Conclusions

Based on the findings of this nationally representative study, sexual minorities experience a high burden of depression and anxiety symptoms compared to heterosexuals. Sexual orientation independently predicts mental health disparities beyond other sociodemographic characteristics. These findings underscore the urgency of addressing mental health inequities among sexual minorities to achieve broader health equity. Targeted interventions and inclusive policies are essential to improve the overall well-being and quality of life for sexual minorities.
